# 4-Nitro-*N*-(4-nitro­benzo­yl)benzamide

**DOI:** 10.1107/S1600536811014450

**Published:** 2011-04-22

**Authors:** Sohail Saeed, Naghmana Rashid, Seik Weng Ng, Edward R. T. Tiekink

**Affiliations:** a Department of Chemistry, Research Complex, Allama Iqbal Open University, Islamabad 44000, Pakistan; b Department of Chemistry, University of Malaya, 50603 Kuala Lumpur, Malaysia

## Abstract

The central acetyl­acetamide moiety in the title compound, C_14_H_9_N_3_O_6_, is buckled [*e.g*. the C—N—C—O torsion angle is 14.3 (6)°] but the r.m.s. deviation for the five atoms is 0.044 Å. The benzene rings lie on the same side of the central plane, forming dihedral angles of 37.17 (15) and 28.58 (19)° with it. The dihedral angle between the two rings is 17.8 (2)° indicating that the mol­ecule is curved. The carbonyl groups are *syn* to each other and *anti* to the amino H atom. This allows for the formation of N—H⋯O hydrogen bonds in the crystal, which leads to twisted chains along the *b* axis. Positional disorder (50:50) of the O atoms was modelled for both the nitro groups.

## Related literature

For background to high-temperature polymers for replacement of ceramics and metals, see: Ataei *et al.* (2005 ▶); Im & Jung, (2000 ▶); Yang *et al.* (2002 ▶).
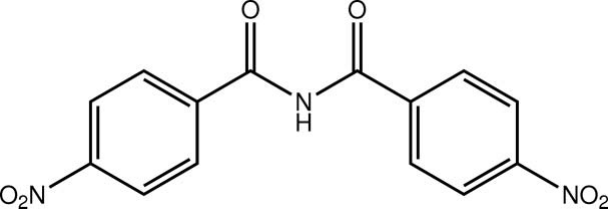


## Experimental

### 

#### Crystal data

C_14_H_9_N_3_O_6_
*M* *_r_* = 315.24
Orthorhombic, 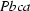

*a* = 13.4757 (7) Å
*b* = 8.5170 (6) Å
*c* = 24.6285 (17) Å
*V* = 2826.7 (3) Å^3^
*Z* = 8
Mo *K*α radiation
μ = 0.12 mm^−1^
*T* = 295 K
0.30 × 0.15 × 0.05 mm

#### Data collection

Agilent Technologies SuperNova Dual diffractometer with an Atlas detector
Absorption correction: multi-scan (*CrysAlis PRO*; Agilent, 2010 ▶) *T*_min_ = 0.965, *T*_max_ = 0.994
13745 measured reflections
2487 independent reflections
1433 reflections with *I* > 2σ(*I*)
*R*_int_ = 0.067

#### Refinement

*R*[*F* ^2^ > 2σ(*F* ^2^)] = 0.065
*wR*(*F* ^2^) = 0.217
*S* = 1.02
2487 reflections
221 parameters
40 restraints
H-atom parameters constrained
Δρ_max_ = 0.38 e Å^−3^
Δρ_min_ = −0.31 e Å^−3^

### 

Data collection: *CrysAlis PRO* (Agilent, 2010 ▶); cell refinement: *CrysAlis PRO*; data reduction: *CrysAlis PRO*; program(s) used to solve structure: *SHELXS97* (Sheldrick, 2008 ▶); program(s) used to refine structure: *SHELXL97* (Sheldrick, 2008 ▶); molecular graphics: *ORTEP-3* (Farrugia, 1997 ▶) and *DIAMOND* (Brandenburg, 2006 ▶); software used to prepare material for publication: *publCIF* (Westrip, 2010 ▶).

## Supplementary Material

Crystal structure: contains datablocks global, I. DOI: 10.1107/S1600536811014450/hb5852sup1.cif

Structure factors: contains datablocks I. DOI: 10.1107/S1600536811014450/hb5852Isup2.hkl

Additional supplementary materials:  crystallographic information; 3D view; checkCIF report

## Figures and Tables

**Table 1:** Hydrogen-bond geometry (Å, °)

*D*—H⋯*A*	*D*—H	H⋯*A*	*D*⋯*A*	*D*—H⋯*A*
N2—H2⋯O3^i^	0.88	2.08	2.951 (4)	170

Symmetry code: (i) 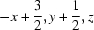.

**Fig. 1.**
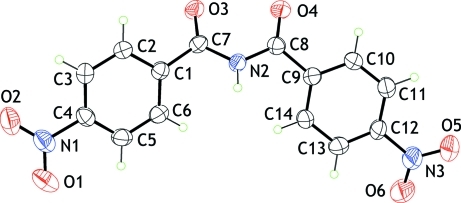
The molecular structure of (I) showing displacement ellipsoids at the 35% probability level.

**Fig. 2.**
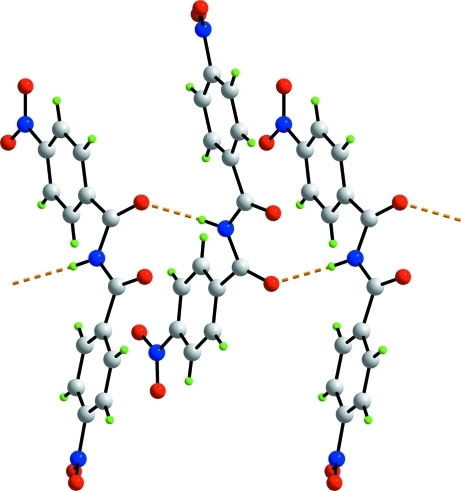
Supramolecular chain aligned along the b axis in (I) mediated by N—H···O hydrogen bonding shown as orange dashed lines.

**Fig. 3.**
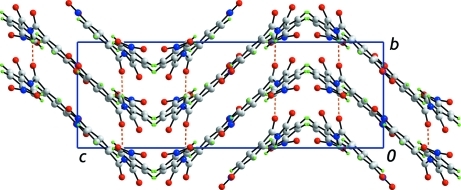
A view in projection down the a axis of the crystal packing in (I), highlighting the undulating layers. The N—H···O hydrogen bonding is shown as orange dashed lines.

## supplementary crystallographic information

### Comment

High-temperature polymers have received much attention owing to interest in the replacement of ceramics and metals (Ataei *et al.*, 2005). However, in many cases they are insoluble and do not melt below their decomposition temperature, a feature that restricts their applications (Im & Jung, 2000). Many studies have therefore focused upon obtaining aromatic polymers that are processable by conventional techniques (Yang *et al.*, 2002). The title compound, (I), is a logical precursor for an attempt to synthesize polyamides and polyimides having excellent thermal and mechanical properties.

Small twists are evident in the central acetylacetamide moiety as seen in the values of the C8—N2—C7—O3 and C7—N2—C8—O4 torsion angles of -4.9 (6) and 14.3 (6) °, respectively. Despite this, the r.m.s. of the fitted atoms from their least-squares plane = 0.0438 Å with the major deviations of 0.0473 (16) and -0.0708 (23) Å being for the O4 and C8 atoms, respectively. The C1- and C9-benzene rings form dihedral angles of 37.17 (15) and 28.58 (19) ° with the central plane, respectively, and form a dihedral angle of 17.8 (2) ° with each other. As the benzene rings lie to the same side of the central plane, overall, the molecule of (I) is curved, Fig. 1. The carbonyl groups are *syn* and the amino-H atom is directed towards the other side of the molecule, *i.e. anti* to the carbonyls. This arrangmement allows for the formation of N—H···O hydorgen bonds leading to a highly twisted chain, Fig. 2 and Table 1. Globally, molecules pack into undulating layers as shown in Fig. 3.

### Experimental

All the reagents and organic solvents were of analytical grade and commercially available. The title compound was accidentally generated during the reaction of 4-nitrobenzoyl chloride with imidazole; it was isolated from the reaction mixture by column chromatography in 45% yield and then purified by re-crystallization from ethanol to give colourless prisms of (I). *M*.pt. 438–439 K; Anal.: C, 53.34; H, 2.88; N, 13.33%. C_14_H_9_N_3_O_6_ requires: C, 53.41; H, 2.87; N, 13.36%.

### Refinement

Carbon-bound H-atoms were placed in calculated positions [C—H 0.93 Å, *U*_iso_(H) 1.2*U*_eq_(C)] and were included in the refinement in the riding model approximation. The amino H-atom was similarly placed [N–H 0.88 Å, *U*_iso_(H) 1.2*U*_eq_(N)].

The nitro groups are disordered over two positions in respect of the O atoms; the disorder could not be refined, and was assumed to be a 1:1 type of disorder. For each group, the N—O distances were restrained to within ±0.01 Å of each other, and the four-atom CNO_2_ unit was restrained to be nearly flat. The displacement parameters were restrained to be nearly isotropic.

### Crystal data

**Table:**

C_14_H_9_N_3_O_6_	*F*(000) = 1296
*M**_r_* = 315.24	*D*_x_ = 1.482 Mg m^−^^3^
Orthorhombic, *P**b**c**a*	Mo *K*α radiation, λ = 0.71073 Å
Hall symbol: -P 2ac 2ab	Cell parameters from 2137 reflections
*a* = 13.4757 (7) Å	θ = 2.4–29.3°
*b* = 8.5170 (6) Å	µ = 0.12 mm^−^^1^
*c* = 24.6285 (17) Å	*T* = 295 K
*V* = 2826.7 (3) Å^3^	Prism, colorless
*Z* = 8	0.30 × 0.15 × 0.05 mm

### Data collection

**Table:**

Agilent Technologies SuperNova Dual diffractometer with an Atlas detector	2487 independent reflections
Radiation source: SuperNova (Mo) X-ray Source	1433 reflections with *I* > 2σ(*I*)
Mirror	*R*_int_ = 0.067
Detector resolution: 10.4041 pixels mm^-1^	θ_max_ = 25.1°, θ_min_ = 3.0°
ω scans	*h* = −16→16
Absorption correction: multi-scan (*CrysAlis PRO*; Agilent, 2010)	*k* = −10→8
*T*_min_ = 0.965, *T*_max_ = 0.994	*l* = −29→29
13745 measured reflections	

### Refinement

**Table:**

Refinement on *F*^2^	Secondary atom site location: difference Fourier map
Least-squares matrix: full	Hydrogen site location: inferred from neighbouring sites
*R*[*F*^2^ > 2σ(*F*^2^)] = 0.065	H-atom parameters constrained
*wR*(*F*^2^) = 0.217	*w* = 1/[σ^2^(*F*_o_^2^) + (0.0936*P*)^2^ + 1.8503*P*] where *P* = (*F*_o_^2^ + 2*F*_c_^2^)/3
*S* = 1.02	(Δ/σ)_max_ = 0.001
2487 reflections	Δρ_max_ = 0.38 e Å^−^^3^
221 parameters	Δρ_min_ = −0.31 e Å^−^^3^
40 restraints	Extinction correction: *SHELXL97* (Sheldrick, 2008), Fc^*^=kFc[1+0.001xFc^2^λ^3^/sin(2θ)]^-1/4^
Primary atom site location: structure-invariant direct methods	Extinction coefficient: 0.0036 (11)

### Fractional atomic coordinates and isotropic or equivalent isotropic displacement parameters (Å^2^)

**Table:**

	*x*	*y*	*z*	*U*_iso_*/*U*_eq_	Occ. (<1)
O1	0.975 (3)	0.868 (3)	0.4716 (10)	0.092 (4)	0.50
O2	1.1108 (6)	0.774 (4)	0.5041 (14)	0.087 (3)	0.50
O1'	0.974 (3)	0.835 (3)	0.4633 (9)	0.092 (4)	0.50
O2'	1.1072 (6)	0.804 (4)	0.5128 (14)	0.087 (3)	0.50
O3	0.84634 (19)	0.2233 (3)	0.64513 (11)	0.0615 (8)	
O4	0.67456 (19)	0.1707 (3)	0.70172 (11)	0.0596 (8)	
O5	0.1877 (10)	0.4334 (12)	0.7162 (4)	0.100 (3)	0.50
O6	0.2086 (12)	0.5041 (11)	0.6323 (4)	0.112 (3)	0.50
O5'	0.1823 (10)	0.3657 (12)	0.6995 (4)	0.100 (3)	0.50
O6'	0.2166 (12)	0.5728 (10)	0.6491 (4)	0.112 (3)	0.50
N1	1.0204 (3)	0.7784 (4)	0.50161 (13)	0.0710 (11)	
N2	0.71486 (19)	0.3916 (4)	0.65368 (12)	0.0489 (8)	
H2	0.6951	0.4882	0.6471	0.059*	
N3	0.2410 (3)	0.4541 (4)	0.67600 (15)	0.0986 (15)	
C1	0.8597 (2)	0.4671 (4)	0.60108 (14)	0.0462 (9)	
C2	0.9620 (3)	0.4820 (5)	0.60666 (15)	0.0518 (10)	
H2A	0.9955	0.4218	0.6323	0.062*	
C3	1.0139 (3)	0.5855 (5)	0.57437 (15)	0.0524 (10)	
H3	1.0821	0.5976	0.5784	0.063*	
C4	0.9630 (3)	0.6702 (4)	0.53628 (14)	0.0525 (10)	
C5	0.8623 (3)	0.6587 (5)	0.52915 (15)	0.0576 (11)	
H5	0.8299	0.7174	0.5027	0.069*	
C6	0.8110 (3)	0.5574 (5)	0.56230 (15)	0.0546 (10)	
H6	0.7425	0.5492	0.5587	0.066*	
C7	0.8080 (3)	0.3493 (4)	0.63559 (15)	0.0468 (9)	
C8	0.6499 (3)	0.2939 (5)	0.68146 (14)	0.0475 (9)	
C9	0.5444 (2)	0.3483 (4)	0.68244 (14)	0.0458 (9)	
C10	0.4802 (3)	0.2851 (5)	0.72057 (15)	0.0578 (11)	
H10	0.5048	0.2180	0.7471	0.069*	
C11	0.3815 (3)	0.3200 (6)	0.71968 (17)	0.0691 (13)	
H11	0.3385	0.2783	0.7454	0.083*	
C12	0.3472 (3)	0.4188 (5)	0.67959 (17)	0.0628 (11)	
C13	0.4080 (3)	0.4851 (5)	0.64193 (17)	0.0628 (11)	
H13	0.3828	0.5532	0.6158	0.075*	
C14	0.5079 (3)	0.4492 (5)	0.64323 (15)	0.0532 (10)	
H14	0.5507	0.4929	0.6177	0.064*	

### Atomic displacement parameters (Å^2^)

**Table:**

	*U*^11^	*U*^22^	*U*^33^	*U*^12^	*U*^13^	*U*^23^
O1	0.125 (3)	0.080 (8)	0.071 (6)	−0.005 (6)	0.007 (5)	0.026 (5)
O2	0.083 (2)	0.087 (9)	0.093 (8)	−0.014 (2)	0.032 (3)	0.006 (5)
O1'	0.125 (3)	0.080 (8)	0.071 (6)	−0.005 (6)	0.007 (5)	0.026 (5)
O2'	0.083 (2)	0.087 (9)	0.093 (8)	−0.014 (2)	0.032 (3)	0.006 (5)
O3	0.0501 (15)	0.0421 (17)	0.092 (2)	0.0075 (12)	0.0066 (14)	0.0080 (14)
O4	0.0601 (17)	0.0487 (17)	0.0699 (17)	0.0045 (13)	0.0018 (13)	0.0090 (14)
O5	0.059 (2)	0.131 (8)	0.109 (6)	0.003 (5)	0.022 (4)	0.021 (5)
O6	0.066 (3)	0.164 (7)	0.105 (5)	0.017 (6)	−0.015 (4)	0.031 (5)
O5'	0.059 (2)	0.131 (8)	0.109 (6)	0.003 (5)	0.022 (4)	0.021 (5)
O6'	0.066 (3)	0.164 (7)	0.105 (5)	0.017 (6)	−0.015 (4)	0.031 (5)
N1	0.088 (3)	0.064 (3)	0.061 (2)	−0.001 (2)	0.018 (2)	0.007 (2)
N2	0.0411 (16)	0.0393 (18)	0.066 (2)	0.0004 (14)	0.0043 (14)	0.0044 (15)
N3	0.054 (2)	0.150 (4)	0.091 (3)	0.011 (3)	0.005 (2)	0.040 (3)
C1	0.045 (2)	0.043 (2)	0.051 (2)	0.0012 (16)	0.0009 (16)	−0.0034 (17)
C2	0.047 (2)	0.055 (2)	0.053 (2)	0.0058 (18)	−0.0012 (17)	0.0064 (19)
C3	0.047 (2)	0.055 (2)	0.055 (2)	−0.0038 (18)	0.0067 (18)	−0.0019 (19)
C4	0.063 (3)	0.049 (2)	0.045 (2)	−0.0002 (19)	0.0097 (18)	0.0019 (18)
C5	0.065 (3)	0.060 (3)	0.048 (2)	0.013 (2)	−0.0016 (19)	0.0076 (19)
C6	0.048 (2)	0.059 (3)	0.057 (2)	0.0057 (19)	−0.0044 (18)	0.001 (2)
C7	0.042 (2)	0.038 (2)	0.060 (2)	0.0013 (17)	−0.0050 (17)	−0.0045 (18)
C8	0.049 (2)	0.044 (2)	0.050 (2)	−0.0030 (18)	−0.0013 (17)	0.0001 (18)
C9	0.044 (2)	0.042 (2)	0.051 (2)	−0.0036 (17)	−0.0047 (17)	−0.0007 (17)
C10	0.054 (2)	0.069 (3)	0.051 (2)	0.0032 (19)	0.0006 (18)	0.014 (2)
C11	0.054 (2)	0.094 (4)	0.059 (3)	0.001 (2)	0.008 (2)	0.016 (2)
C12	0.037 (2)	0.087 (3)	0.064 (3)	0.000 (2)	0.0038 (19)	0.012 (2)
C13	0.052 (2)	0.073 (3)	0.063 (3)	0.003 (2)	−0.009 (2)	0.015 (2)
C14	0.046 (2)	0.056 (2)	0.058 (2)	−0.0063 (18)	0.0020 (18)	0.0086 (19)

### Geometric parameters (Å, °)

**Table:**

O1—N1	1.228 (7)	C2—H2A	0.9300
O2—N1	1.221 (7)	C3—C4	1.367 (5)
O1'—N1	1.228 (7)	C3—H3	0.9300
O2'—N1	1.222 (7)	C4—C5	1.372 (5)
O3—C7	1.214 (4)	C5—C6	1.374 (5)
O4—C8	1.209 (4)	C5—H5	0.9300
O5—N3	1.235 (7)	C6—H6	0.9300
O6—N3	1.237 (7)	C8—C9	1.496 (5)
O5'—N3	1.236 (7)	C9—C14	1.383 (5)
O6'—N3	1.254 (7)	C9—C10	1.385 (5)
N1—C4	1.475 (5)	C10—C11	1.363 (5)
N2—C7	1.380 (4)	C10—H10	0.9300
N2—C8	1.388 (4)	C11—C12	1.377 (6)
N2—H2	0.8800	C11—H11	0.9300
N3—C12	1.466 (5)	C12—C13	1.360 (6)
C1—C2	1.391 (5)	C13—C14	1.381 (5)
C1—C6	1.391 (5)	C13—H13	0.9300
C1—C7	1.488 (5)	C14—H14	0.9300
C2—C3	1.378 (5)		
			
O2—N1—O1	123 (3)	C4—C5—H5	121.1
O2'—N1—O1'	126 (3)	C6—C5—H5	121.1
O2—N1—C4	118.4 (19)	C5—C6—C1	121.2 (4)
O2'—N1—C4	118.9 (19)	C5—C6—H6	119.4
O1—N1—C4	118 (2)	C1—C6—H6	119.4
O1'—N1—C4	115 (2)	O3—C7—N2	123.7 (3)
C7—N2—C8	125.2 (3)	O3—C7—C1	120.5 (3)
C7—N2—H2	117.4	N2—C7—C1	115.7 (3)
C8—N2—H2	117.4	O4—C8—N2	123.4 (3)
O5—N3—O6	122.7 (12)	O4—C8—C9	121.6 (3)
O5—N3—O6'	112.7 (10)	N2—C8—C9	115.0 (3)
O5'—N3—O6'	124.8 (12)	C14—C9—C10	119.5 (3)
O5—N3—C12	119.4 (8)	C14—C9—C8	121.2 (3)
O6—N3—C12	117.9 (9)	C10—C9—C8	119.0 (3)
O5'—N3—C12	118.2 (8)	C11—C10—C9	120.9 (4)
O6'—N3—C12	117.0 (9)	C11—C10—H10	119.5
C2—C1—C6	119.0 (3)	C9—C10—H10	119.5
C2—C1—C7	118.0 (3)	C10—C11—C12	118.2 (4)
C6—C1—C7	123.0 (3)	C10—C11—H11	120.9
C3—C2—C1	120.3 (3)	C12—C11—H11	120.9
C3—C2—H2A	119.9	C13—C12—C11	122.7 (4)
C1—C2—H2A	119.9	C13—C12—N3	117.5 (4)
C4—C3—C2	118.6 (4)	C11—C12—N3	119.8 (3)
C4—C3—H3	120.7	C12—C13—C14	118.6 (4)
C2—C3—H3	120.7	C12—C13—H13	120.7
C3—C4—C5	123.1 (3)	C14—C13—H13	120.7
C3—C4—N1	117.7 (4)	C13—C14—C9	120.0 (3)
C5—C4—N1	119.3 (3)	C13—C14—H14	120.0
C4—C5—C6	117.8 (3)	C9—C14—H14	120.0
			
C6—C1—C2—C3	0.4 (5)	C7—N2—C8—O4	14.3 (6)
C7—C1—C2—C3	177.8 (3)	C7—N2—C8—C9	−162.8 (3)
C1—C2—C3—C4	−1.3 (6)	O4—C8—C9—C14	−153.9 (4)
C2—C3—C4—C5	1.0 (6)	N2—C8—C9—C14	23.3 (5)
C2—C3—C4—N1	−179.0 (3)	O4—C8—C9—C10	20.4 (5)
O2—N1—C4—C3	8.3 (14)	N2—C8—C9—C10	−162.4 (3)
O2'—N1—C4—C3	−9.7 (14)	C14—C9—C10—C11	0.6 (6)
O1—N1—C4—C3	−171.8 (14)	C8—C9—C10—C11	−173.8 (4)
O1'—N1—C4—C3	170.3 (14)	C9—C10—C11—C12	0.5 (6)
O2—N1—C4—C5	−171.7 (14)	C10—C11—C12—C13	−1.5 (7)
O2'—N1—C4—C5	170.3 (14)	C10—C11—C12—N3	177.6 (4)
O1—N1—C4—C5	8.2 (14)	O5—N3—C12—C13	−160.6 (6)
O1'—N1—C4—C5	−9.7 (14)	O6—N3—C12—C13	19.3 (6)
C3—C4—C5—C6	0.3 (6)	O5'—N3—C12—C13	161.1 (6)
N1—C4—C5—C6	−179.7 (3)	O6'—N3—C12—C13	−18.9 (6)
C4—C5—C6—C1	−1.3 (6)	O5—N3—C12—C11	20.2 (6)
C2—C1—C6—C5	1.0 (6)	O6—N3—C12—C11	−159.9 (6)
C7—C1—C6—C5	−176.3 (3)	O5'—N3—C12—C11	−18.1 (6)
C8—N2—C7—O3	−4.9 (6)	O6'—N3—C12—C11	161.9 (6)
C8—N2—C7—C1	173.0 (3)	C11—C12—C13—C14	1.4 (7)
C2—C1—C7—O3	−38.2 (5)	N3—C12—C13—C14	−177.7 (4)
C6—C1—C7—O3	139.1 (4)	C12—C13—C14—C9	−0.3 (6)
C2—C1—C7—N2	143.8 (3)	C10—C9—C14—C13	−0.7 (6)
C6—C1—C7—N2	−38.9 (5)	C8—C9—C14—C13	173.5 (4)

### Hydrogen-bond geometry (Å, °)

**Table:**

*D*—H···*A*	*D*—H	H···*A*	*D*···*A*	*D*—H···*A*
N2—H2···O3^i^	0.88	2.08	2.951 (4)	170

Symmetry codes: (i) −*x*+3/2, *y*+1/2, *z*.
